# Case Report: 26-year follow-up after total cavopulmonary connection in crisscross heart—longitudinal assessment of anastomotic patency, biomarkers, and arrhythmia progression

**DOI:** 10.3389/fcvm.2026.1860212

**Published:** 2026-07-28

**Authors:** Xiaohui Zhang, Abulipizi Abudukadier, Haining Zheng, Chaoyang Wen

**Affiliations:** Department of Ultrasound, Peking University International Hospital, Beijing, China

**Keywords:** adult congenital heart disease, anastomotic patency, atrial arrhythmia, crisscross heart, fontan circulation, long-term follow-up, NT-ProBNP, total cavopulmonary connection

## Abstract

Crisscross heart (CCH) is an extremely rare congenital cardiac malformation, and literature on long-term outcomes after total cavopulmonary connection (TCPC) in CCH patients remains limited. We report a unique case demonstrating sustained anastomotic patency and objective postoperative evolution 26 years after TCPC in a patient with CCH. A 39-year-old female with crisscross heart and double-outlet right ventricle underwent TCPC at age 13 in 2000. Serial Doppler echocardiography (2013–2026) consistently demonstrated patent superior vena cava–right pulmonary artery anastomosis and inferior vena cava conduit without obstructive flow velocities (SVC-RPA: 30.6–43.9 cm/s; IVC conduit: 39.0–23.2 cm/s). The clinical course was complicated by progressive atrial arrhythmias: first-degree atrioventricular block with atrial premature beats (2017), atrial fibrillation with incomplete right bundle branch block (2020), and atypical atrial flutter (2026). NT-proBNP increased from 124 pg/mL (2024) to 592.3 pg/mL (2026), with persistently elevated hemoglobin (164–171 g/L). Management included beta-blockers for rate control and clinical surveillance without anticoagulation. At 26 years post-TCPC, the patient maintained NYHA functional class II, with patent Fontan pathways and preserved left ventricular function (EF 60%, 2026), and no protein-losing enteropathy, plastic bronchitis, or thromboembolic events. This case demonstrates that durable anastomotic patency and preserved functional status are achievable, while highlighting the inevitability of progressive atrial arrhythmias necessitating lifelong surveillance.

## 1 Introduction

Crisscross heart (CCH) is a rare congenital cardiac anomaly characterized by atrioventricular connection twisting and ventricular inflow crossing (1). The technical feasibility of Fontan-type procedures in CCH has been described; however, detailed case-level follow-up data beyond two decades in patients with complex underlying anatomy remain scarce. Since the original description of the Fontan procedure for tricuspid atresia (2) and the subsequent evolution to total cavopulmonary connection (TCPC) (3), long-term outcomes have progressively improved, with contemporary series reporting 20-year survival rates of approximately 80% (4). Most published CCH case reports focus on preoperative diagnosis and early surgical outcomes, while longitudinal multimodality follow-up—particularly Doppler echocardiographic documentation of cavopulmonary anastomotic patency—remains limited in the literature.

The present report offers three principal contributions. First, we demonstrate sustained cavopulmonary anastomotic patency through serial Doppler echocardiography from 2013 to 2026, providing direct hemodynamic evidence of conduit durability. Second, we objectively document the longitudinal evolution of biomarkers (NT-proBNP) and cardiac rhythm. Third, through evaluation of the patient's current status without anticoagulation, we highlight potential gaps in anticoagulation management among adults with Fontan circulation (5).

This report aims to present a 26-year longitudinal follow-up of a CCH patient after TCPC, providing detailed serial data encompassing echocardiography, electrocardiography, and laboratory parameters to inform clinical surveillance strategies for similar patients.

## 2 Case description

A 39-year-old female presented with cyanosis since birth and was diagnosed during childhood with crisscross heart (CCH) complicated by double-outlet right ventricle (DORV), ventricular septal defect (VSD), and pulmonary valve stenosis. She underwent TCPC surgery at an external hospital at age 13 in 2000.

The patient first visited our center's cardiac surgery outpatient clinic in September 2025, with a resting oxygen saturation of 96% and unremarkable physical examination. The visit diagnosis was postoperative congenital heart disease in a stable clinical state. In April 2026, she returned with a 6-month history of intermittent palpitations. Cardiac auscultation revealed arrhythmia, clinically suspected as atrial fibrillation; notably, medical records clearly documented the absence of anticoagulant use. On the same day, she also visited the emergency department with complaints of intermittent palpitations for 6 months, occasional chest and back pain, and tachycardia upon exertion. Emergency ECG recorded atypical atrial flutter, and physical examination revealed pulse deficit (peripheral pulse rate lower than heart rate). Based on the patient's ability to perform daily physical activities with only mild symptoms and no significant limitation, her functional status was classified as NYHA class II.

Regarding past medical history, serial electrocardiograms documented progressive atrial arrhythmias, including first-degree atrioventricular block with atrial premature beats (2017), atrial fibrillation with incomplete right bundle branch block (QRS axis +120°) (2020), and atypical atrial flutter (QRS axis +191°) (2026). Throughout the follow-up period, the patient experienced no heart failure hospitalizations, thromboembolic events, protein-losing enteropathy, or plastic bronchitis.

### 2.1 Clinical timeline

**Figure 1:**
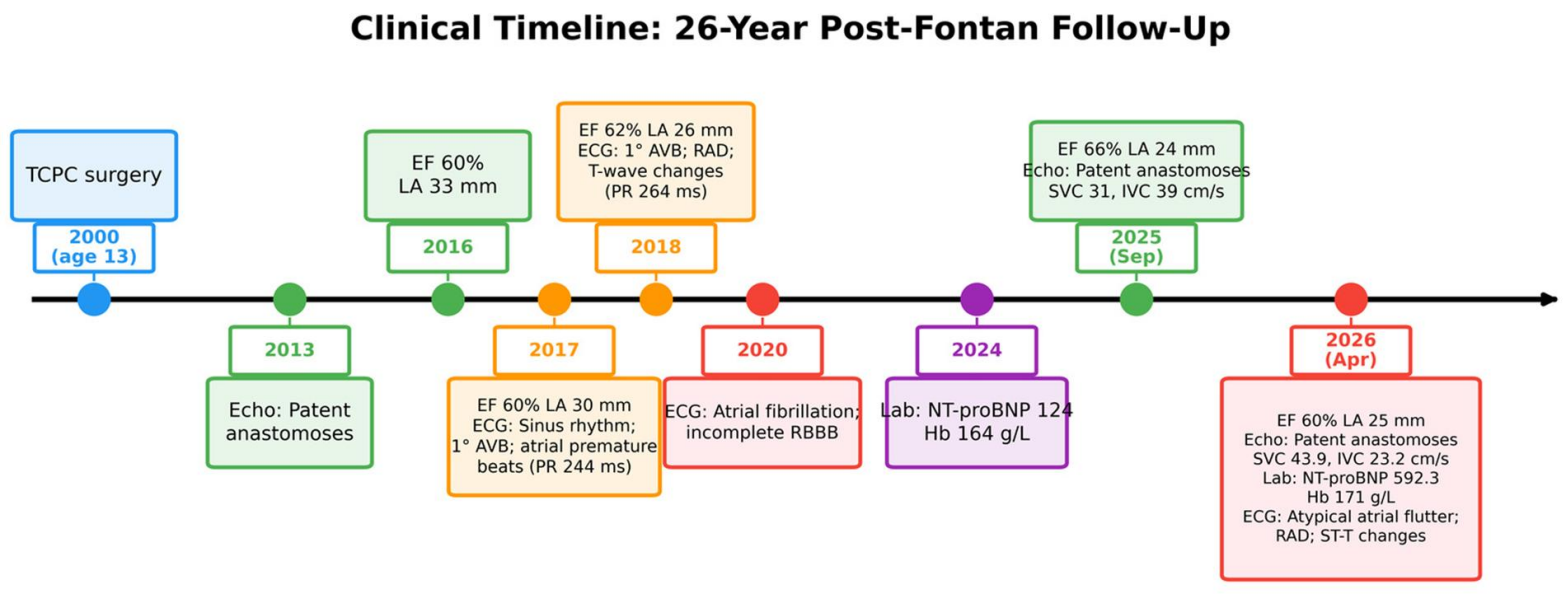
Clinical timeline of 26-year post-Fontan follow-up. The timeline illustrates key clinical events from TCPC surgery in 2000 (age 13) to the most recent follow-up in 2026 (age 39), including serial echocardiographic assessments (green), electrocardiographic rhythm evolution (orange/red), and laboratory biomarker measurements (purple). Color coding: Blue=surgery; Green=echocardiography; Orange/Red=ECG rhythm progression; Purple=laboratory.

Figure 1 presents a comprehensive clinical timeline from the TCPC surgery in 2000 to the most recent follow-up in 2026, summarizing key echocardiographic, electrocardiographic, and laboratory milestones.

## 3 Diagnostic assessment

### 3.1 Doppler assessment of cavopulmonary anastomotic patency

**Figure 2:**
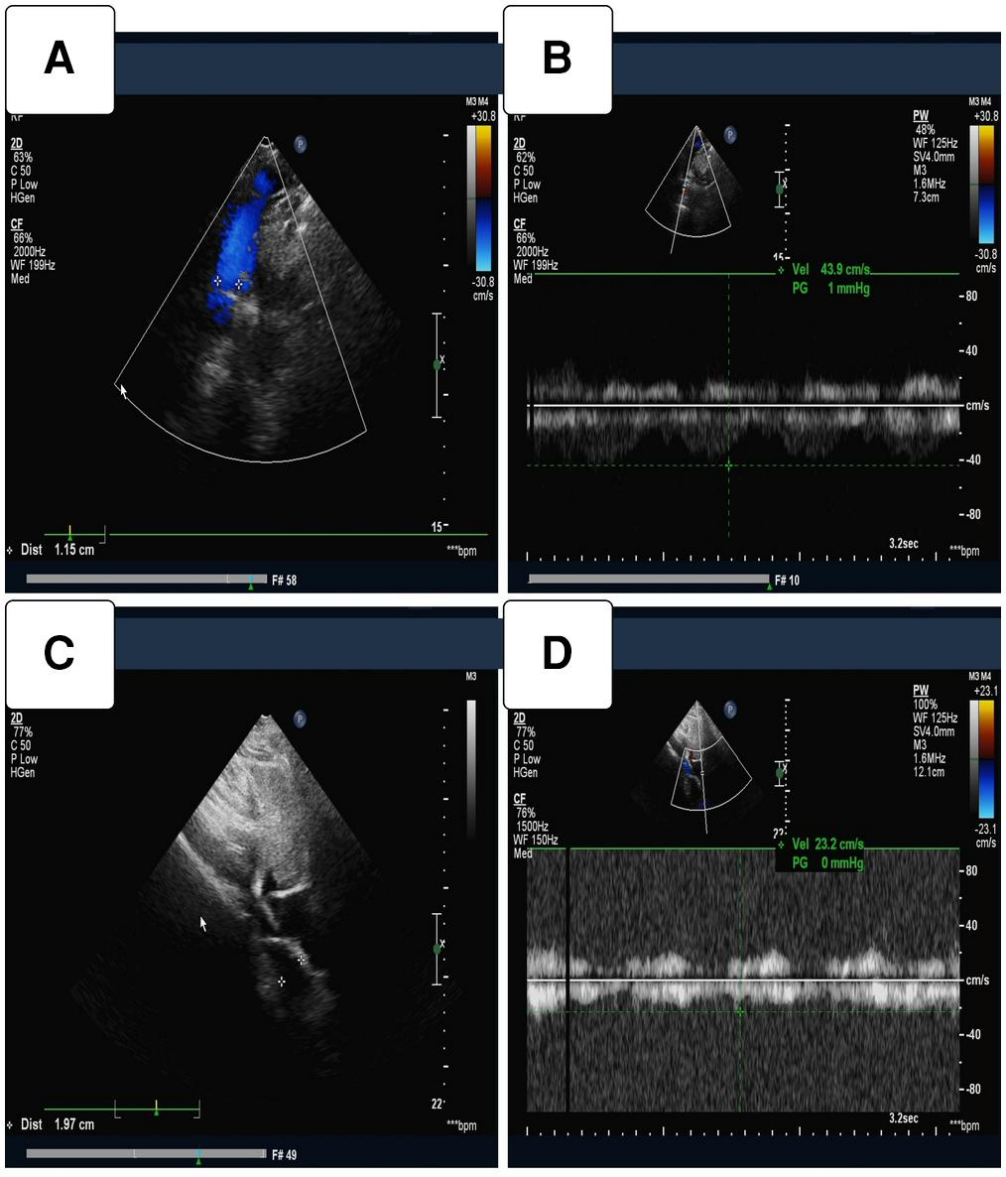
Transthoracic echocardiography in April 2026 (26 years post-TCPC). **(A)** Color Doppler imaging of the SVC-RPA anastomosis demonstrating patency with a diameter of approximately 12 mm. **(B)** Pulsed-wave Doppler of the SVC-RPA anastomosis showing unobstructed flow velocity of 43.9 cm/s. **(C)** Two-dimensional grayscale imaging of the IVC conduit showing a diameter of approximately 19 mm. **(D)** Pulsed-wave Doppler of the IVC conduit showing unobstructed flow velocity of 23.2 cm/s.

Transthoracic echocardiography (TTE) consistently demonstrated patent superior vena cava–right pulmonary artery (SVC-RPA) anastomosis and patent inferior vena cava (IVC) conduit without obstructive flow patterns. The most recent TTE evaluation (April 2026) is shown in Figure 2. Left ventricular ejection fraction (LVEF) remained normal throughout follow-up (60%–66%), and left atrial diameter progressively decreased from 33 mm (2016) to 25 mm (2026), suggesting stable hemodynamic status.

### 3.2 Laboratory findings

Laboratory evaluation included serial measurements of N-terminal pro-B-type natriuretic peptide (NT-proBNP), complete blood count, coagulation parameters, and liver function. NT-proBNP, a prognostic biomarker in Fontan circulation (6), increased from 124 pg/mL (2024) to 592.3 pg/mL (2026), suggesting evolving hemodynamic stress despite preserved left ventricular systolic function. Hemoglobin remained persistently elevated (164–171 g/L) with hematocrit of 49.2%–51.1%, consistent with compensatory changes in Fontan physiology. Prothrombin time (12.2–13.0 s) and international normalized ratio (1.05–1.19) were within normal ranges. Direct bilirubin increased mildly from 6.7 to 8.1 μmol/L, potentially reflecting early Fontan-associated hepatic congestion or chronic hemolysis (Table 1). The differential diagnosis for NT-proBNP elevation included worsening venous congestion in the Fontan circulation, increased atrial arrhythmia burden, and early hepatic congestion, all of which were considered in the ongoing management strategy.

**Table 1:** Serial laboratory and electrocardiographic findings in the post-Fontan patient.

A. Laboratory parameters		
Parameter	2024	2026 April
NT-proBNP (pg/mL)	124	592.3
Hemoglobin (g/L)	164	171
Hematocrit (%)	49.2	51.1
Prothrombin time (s)	12.2	13.0
INR	1.05	1.19
Direct bilirubin (*μ*mol/L)	6.7	8.1

B. Electrocardiographic evolution				
Parameter	2017	2018	2020	2026 April
Heart rate (bpm)	73	82	72	126
PR interval (ms)	244	264	—	—
QTc (ms)	414	457	433	477
QRS axis	+29°	RAD	+120°	+191°
Rhythm	Sinus rhythm/ 1°AVB/APBs	1°AVB/RAD/ T wave changes	AF/incomplete RBBB/T wave changes	Atypical atrial flutter/ RAD/V1 R/s > 1/ ST-T changes

NT-proBNP, N-terminal pro-B-type natriuretic peptide; INR, international normalized ratio; AVB, atrioventricular block; APBs, atrial premature beats; RAD, right axis deviation; AF, atrial fibrillation; RBBB, right bundle branch block.

### 3.3 Electrocardiographic evolution

Arrhythmia progression is a well-recognized complication in the Fontan population (7). The ECG evolution demonstrated a clear temporal trajectory of increasing arrhythmia burden: sinus rhythm with first-degree atrioventricular block (PR interval 244 ms) and atrial premature beats were recorded in 2017; PR interval prolongation to 264 ms, persistent right axis deviation, and T-wave changes appeared in 2018; atrial fibrillation with incomplete right bundle branch block and a QRS axis of +120° emerged in 2020; and the rhythm evolved to atypical atrial flutter in 2026 with a QRS axis of +191° (Table 1). This progressive arrhythmia evolution is consistent with the known natural history of Fontan circulation, in which the prevalence of atrial arrhythmias increases with longer follow-up duration.

## 4 Treatment, follow-up, and outcomes

The patient has a history of allergy to Qingkailing (a Chinese herbal injection). Current medications include hydrochlorothiazide 25 mg once daily, spironolactone 20 mg once daily, potassium chloride 0.5 g once daily, atenolol 25 mg once daily, and metoprolol 12.5 mg once daily. This regimen is consistent with contemporary guidelines for arrhythmia management in adult congenital heart disease (8).

Of particular note, the patient is currently not receiving any anticoagulant therapy. She was prescribed aspirin postoperatively but discontinued it due to intolerable menorrhagia; no alternative anticoagulation (e.g., warfarin) was subsequently initiated. This decision merits discussion. Current guidelines recommend considering anticoagulation for Fontan patients with atrial arrhythmias, given the elevated thromboembolic risk inherent to Fontan circulation (5, 9). The absence of anticoagulation in this patient highlights a potential gap in the management of adult Fontan survivors.

The patient has maintained NYHA functional class II for many years, independently performing daily activities and continuing to work. A slow, mild decline in exercise tolerance in recent years is a recognized pattern in the aging Fontan population. The cardiac surgery outpatient clinic at our center documented stable clinical status with oxygen saturation of 96% in September 2025. In April 2026, the patient presented with a 6-month history of intermittent palpitations and occasional chest and back pain with exertional tachycardia. ECG documented atrial premature beats with a clinical impression of atrial fibrillation, consistent with the serial ECG-recorded arrhythmia evolution (Table 1). Throughout follow-up, the patient has experienced no heart failure hospitalizations, thromboembolic events, protein-losing enteropathy, or plastic bronchitis. The overall course is that of a stable long-term Fontan survivor with durable surgical benefit and consistently patent anastomoses confirmed by serial Doppler studies, though progressive atrial arrhythmia has become the primary clinical concern.

## 5 Discussion

### 5.1 Strengths and limitations

The strength of this report lies in the availability of longitudinal follow-up data spanning 26 years after TCPC, including serial Doppler assessments, biomarkers, and ECG monitoring, providing valuable reference for long-term management of adult Fontan patients. However, limitations include incomplete clinical data at certain key time points due to patient follow-up compliance, precluding a complete assessment of the continuous trajectory of Fontan circulation evolution. Additionally, the absence of cardiac magnetic resonance (CMR) data is a significant limitation; CMR is the gold standard for assessing Fontan conduit hemodynamics, ventricular volumes and function, and hepatic congestion, and its absence constrains precise evaluation of subclinical Fontan failure and Fontan-associated liver disease (FALD) progression (10). Similarly, the lack of exercise stress testing or cardiopulmonary exercise testing (CPET) precludes objective quantification of the patient's declining exercise tolerance and its correlation with cardiac function parameters.

### 5.2 Comparison with the literature

The findings in this case are both consistent with and complementary to the existing literature on long-term Fontan outcomes. Survival of 26 years with NYHA class II functional status aligns with data from large Fontan registries. The Australian and New Zealand Fontan Registry reported 20-year survival of approximately 80% after Fontan surgery, with the majority of survivors maintaining favorable functional status (11). Similarly, d'Udekem et al. reported substantially improved 25-year survival with modern surgical techniques, though late complications remain ongoing challenges (4).

### 5.3 Lessons learned

The absence of anticoagulation in this patient, despite the presence of Fontan circulation-associated thrombotic risk and a history of atrial fibrillation/flutter, warrants attention. This case highlights a potential gap in anticoagulation management among adult Fontan patients. Current evidence suggests that anticoagulation should be strongly considered to reduce thromboembolic risk in this population (9, 12, 13). The decision to withhold anticoagulation must balance the thrombotic risk against bleeding risk; in this patient, the history of aspirin-induced menorrhagia presented a specific challenge. This case underscores the need for individualized anticoagulation strategies in Fontan patients with atrial arrhythmias.

## 6 Patient perspective

The patient reported having cyanosis since birth, with purple discoloration of the lips, fingernails, and toenails, accompanied by clubbing. She experienced occasional hemoptysis during winter months. In recent years, she has developed palpitations and tachycardia. She was evaluated at a tertiary cardiac center for possible implantable cardioverter-defibrillator (ICD) implantation or catheter ablation, but the procedures were not performed due to unsuitable vascular anatomy. In April 2026, she presented to the emergency department with a 6-month history of intermittent palpitations and occasional chest and back pain with exertional tachycardia. Emergency physicians detected a pulse deficit, though her general condition was stable. She currently experiences mildly decreased exercise tolerance compared with her previous capacity.

## Data Availability

The original contributions presented in the study are included in the article/Supplementary Material, further inquiries can be directed to the corresponding author.
